# Supplementary material for: Implementation of E-exams during the COVID-19 pandemic: A quantitative study in higher education
Source: PLoS One. 2022 May 20;17(5):e0266940. doi: 10.1371/journal.pone.0266940 (PMC9122221; doi:10.1371/journal.pone.0266940)
Supplement: Attachment — Submitted filename: PONE-D-21-20789_reviewer.pdf [file pone.0266940.s002.pdf]

PLOS ONE

Implementation of electronic exams during the spread of the COVID-19 pandemic: A quantitative study in higher education

--Manuscript Draft--

**Manuscript Number:**	PONE-D-21-20789
**Article Type:**	Research Article
**Full Title:**	Implementation of electronic exams during the spread of the COVID-19 pandemic: A quantitative study in higher education
**Short Title:**	E- exams during the spread of the COVID-19 pandemic: A quantitative study in higher education
**Corresponding Author:**	Najeh Rajeh Alsalhi, Ph.D<br>Ajman University<br>Ajman, --- Select One --- UNITED ARAB EMIRATES
**Keywords:**	Electronic Exams; perspective; undergraduate students; Higher education; COVID-19 pandemic
**Abstract:**	The primary aim of this study was to identify the degree of acceptance of electronic exams by undergraduate students at Ajman University during the spread of the COVID-19 pandemic. The study used the descriptive approach. A questionnaire consisting of 27 items was distributed to 1986 undergraduate students. The results of the study showed that undergraduate students demonstrated a moderate degree of acceptance of the implementation of electronic exams during the spread of the COVID-19 pandemic, with females students finding them more acceptable than male students. Discipline and academic year also showed an impact on such acceptance, with Pharmacy & Health Science College students, and those in their third academic year demonstrating the highest levels of acceptance. Implications of the study raise awareness of the importance of addressing challenges associated with electronic exams such as strict computer technology settings.
**Order of Authors:**	Mohd. Elmagzoub Eltahir
	Najeh Rajeh Alsalhi, Ph.D
	Sami Sulieman Al-Qatawneh
**Additional Information:**	
**Question**	**Response**
**Financial Disclosure**<br><br>Enter a financial disclosure statement that describes the sources of funding for the work included in this submission. Review the submission guidelines for detailed requirements. View published research articles from *PLOS ONE* for specific examples.<br><br>This statement is required for submission and **will appear in the published article** if the submission is accepted. Please make sure it is accurate.	The author(s) received no specific funding for this work.

Powered by Editorial Manager® and ProduXion Manager® from Aries Systems Corporation

**Unfunded studies**<br>Enter: *The author(s) received no specific funding for this work.*<br><br>**Funded studies**<br>Enter a statement with the following details:<br>• Initials of the authors who received each award<br>• Grant numbers awarded to each author<br>• The full name of each funder<br>• URL of each funder website<br>• Did the sponsors or funders play any role in the study design, data collection and analysis, decision to publish, or preparation of the manuscript?<br>• **NO** - Include this sentence at the end of your statement: *The funders had no role in study design, data collection and analysis, decision to publish, or preparation of the manuscript.*<br>• **YES** - Specify the role(s) played.<br><br>* typeset	
**Competing Interests**<br><br>Use the instructions below to enter a competing interest statement for this submission. On behalf of all authors, disclose any competing interests that could be perceived to bias this work—acknowledging all financial support and any other relevant financial or non-financial competing interests.<br><br>This statement is required for submission and **will appear in the published article** if the submission is accepted. Please make sure it is accurate and that any funding sources listed in your Funding Information later in the submission form are also declared in your Financial Disclosure statement.<br><br>View published research articles from *PLOS ONE* for specific examples.	The authors have declared that no competing interests exist

**NO authors have competing interests**<br><br>Enter: *The authors have declared that no competing interests exist.*<br><br>**Authors with competing interests**<br><br>Enter competing interest details beginning with this statement:<br><br>*I have read the journal's policy and the authors of this manuscript have the following competing interests: [insert competing interests here]*<br><br>* typeset	
**Ethics Statement**<br><br>Enter an ethics statement for this submission. This statement is required if the study involved:<br><br>• Human participants<br>• Human specimens or tissue<br>• Vertebrate animals or cephalopods<br>• Vertebrate embryos or tissues<br>• Field research<br><br>Write "N/A" if the submission does not require an ethics statement.<br><br>General guidance is provided below. Consult the submission guidelines for detailed instructions. **Make sure that all information entered here is included in the Methods section of the manuscript.**	- The Research Ethics Committee - Deanship of Graduate Studies and Research- Ajman University.<br>- Reference Number: H-H-F-2020-May-28<br>- The form of consent obtained (written)

Powered by Editorial Manager® and ProduXion Manager® from Aries Systems Corporation

**Format for specific study types**<br><br>**Human Subject Research (involving human participants and/or tissue)**<br>• Give the name of the institutional review board or ethics committee that approved the study<br>• Include the approval number and/or a statement indicating approval of this research<br>• Indicate the form of consent obtained (written/oral) or the reason that consent was not obtained (e.g. the data were analyzed anonymously)<br><br>**Animal Research (involving vertebrate animals, embryos or tissues)**<br>• Provide the name of the Institutional Animal Care and Use Committee (IACUC) or other relevant ethics board that reviewed the study protocol, and indicate whether they approved this research or granted a formal waiver of ethical approval<br>• Include an approval number if one was obtained<br>• If the study involved *non-human primates*, add *additional details* about animal welfare and steps taken to ameliorate suffering<br>• If anesthesia, euthanasia, or any kind of animal sacrifice is part of the study, include briefly which substances and/or methods were applied<br><br>**Field Research**<br><br>Include the following details if this study involves the collection of plant, animal, or other materials from a natural setting:<br>• Field permit number<br>• Name of the institution or relevant body that granted permission	
**Data Availability**<br><br>Authors are required to make all data underlying the findings described fully available, without restriction, and from the time of publication. PLOS allows rare exceptions to address legal and ethical concerns. See the PLOS Data Policy and FAQ for detailed information.	Yes - all data are fully available without restriction

A Data Availability Statement describing where the data can be found is required at submission. Your answers to this question constitute the Data Availability Statement and **will be published in the article**, if accepted.<br><br>**Important:** Stating 'data available on request from the author' is not sufficient. If your data are only available upon request, select 'No' for the first question and explain your exceptional situation in the text box.<br><br>Do the authors confirm that all data underlying the findings described in their manuscript are fully available without restriction?	
**Describe where the data may be found in full sentences. If you are copying our sample text, replace any instances of XXX with the appropriate details.**<br><br>• If the data are **held or will be held in a public repository**, include URLs, accession numbers or DOIs. If this information will only be available after acceptance, indicate this by ticking the box below. For example: *All XXX files are available from the XXX database (accession number(s) XXX, XXX.).*<br>• If the data are all contained **within the manuscript and/or Supporting Information files**, enter the following: *All relevant data are within the manuscript and its Supporting Information files.*<br>• If neither of these applies but you are able to provide **details of access elsewhere**, with or without limitations, please do so. For example:<br><br>*Data cannot be shared publicly because of [XXX]. Data are available from the XXX Institutional Data Access / Ethics Committee (contact via XXX) for researchers who meet the criteria for access to confidential data.*<br><br>*The data underlying the results presented in the study are available from (include the name of the third party*	All relevant data are within the manuscript and its Supporting Information files.

and contact information or URL).<br>• This text is appropriate if the data are owned by a third party and authors do not have permission to share the data.<br><br>* typeset	
Additional data availability information:	

Manuscript Click here to access/download;Manuscript;Manuscript.docx

Implementation of electronic exams during the spread of the COVID-19 pandemic: A quantitative study in higher education

Mohd. Elmagzoub Eltahir [1, 2, a]

m.babiker@ajman.ac.ae

Najeh Rajeh Alsalhi [1, 2, b]*

n.alsalhi@ajman.ac.ae

(00971543374987)

Sami Sulieman Al-Qatawneh [1, c]

s.alqatawneh@ajman.ac.ae

[1]*College of Humanities and Sciences, Ajman University, Ajman, UAE*
[2]*Nonlinear Dynamics Research Center (NDRC), Ajman University, Ajman, UAE*

*Corresponding author details

Najeh Rajeh Alsalhi

n.alsalhi@ajman.ac.ae

Abstract

The primary aim of this study was to identify the degree of acceptance of electronic exams by undergraduate students at Ajman University during the spread of the COVID-19 pandemic. The study used the descriptive approach. A questionnaire consisting of 27 items was distributed to 1986 undergraduate students. The results of the study showed that undergraduate students demonstrated a moderate degree of acceptance of the implementation of electronic exams during the spread of the COVID-19 pandemic, with females students finding them more acceptable than male students. Discipline and academic year also showed an impact on such acceptance, with Pharmacy & Health Science College students, and those in their third academic year demonstrating the highest levels of acceptance. Implications of the study raise awareness of the importance of addressing challenges associated with electronic exams such as strict computer technology settings.

Keywords*:* Electronic Exams, perspective, undergraduate students, Higher education, COVID-19 pandemic.

1. Introduction

The education system is witnessing great and successive developments to keep pace with the changes resulting from the advancement of science and technology and the implications of the spread of the COVID-19 pandemic. During the past few months, most national governments have shut down in-person teaching in their educational institutions completely or partially to curb the rapid spread of COVID-19 (Giannini, Jenkins, & Saavedra, 2020). Educational institutions and systems have therefore sought to adapt to these developments through teaching and evaluation strategies suitable to this new environment of the COVID-19 pandemic.

The evaluation process is among the main components of any stage of the educational process, because through it students are sorted according to their abilities and their achievement progress. Sutton (2004) indicates that improving and developing evaluation methods is one of the five goals of the academic community, as international academic accreditation institutions such as the North Central Association of Colleges and Schools (NCA) and the National Council for Accreditation of Teacher Education (NCATE) consider evaluation as one of the basic and necessary criteria for accreditation. Electronic exams came to provide a great service to the education system during the spread of a COVID-19 pandemic. Electronic exams also represent other benefits. They save time and effort for faculty members, and are safer for students in comparison to traditional printed paper tests: the latter require more time and effort to correct them and extract results and announce them to students, whereas with the former, all this effort is made by the computer quickly and accurately (Ahmed, 2004). In addition, electronic exams are considered one of the most important e-learning tools that measure achievement in developed countries. Technology has enabled modern, unconventional evaluation methods, such as computerized evaluation, online assessment, remote evaluation, and question banks. Nowadays, during the COVID-19 pandemic, the electronic exams system becomes looked to be a rapidly developing assessment instrument due to its precision and reliability.

According to Abass, Olajide and Samuel (2017), most educational institutions started using the electronic exams system during the COVID-19 pandemic due to its positive features, such as reductions in the time required for students' exams and institutions easily monitoring students

during their examination. However, students' perspectives on its implementation in Emirati universities such as Ajman University remain unexplored. Thus, the study sought to investigate the implementation of electronic exams during the COVID-19 pandemic in higher education institutions in the UAE. Electronic exams were carried out at Ajman University in the UAE, in the fall of 2020 during the spread of COVID-19. The current study is therefore aimed at investigating students' acceptance of the implementation of electronic exams in their university.

The authors hope this investigation will help officials in higher education institutions and universities to make appropriate decisions about the permanent adoption of electronic exams during the COVID-19 pandemic and the possibility of their application after the pandemic. An addition aim is to give university officials feedback on the level of students' acceptance of electronic exams during the COVID-19 pandemic. This means that the current study may supply higher education institutions with sufficient information about students' degree of acceptance of the electronic exams implemented during the spread of the COVID-19 pandemic. In turn, this will assist in the adoption of electronic exams as a reliable assessment instrument, and a valid alternative to traditional printed examinations in higher education institutions in the future.

In order to explore Ajman University undergraduate students' degree of acceptance of electronic exams during the spread of the COVID-19 pandemic, the study seeks to answers to the following research questions:

RQ1: To what extent do Ajman University undergraduate students accept electronic exams during the spread of the COVID-19 pandemic?

RQ2: Does the degree of Ajman University undergraduate students' acceptance of electronic exams during the spread of COVID-19 vary according to gender, college, and academic year?

2. Literature Review

2.1 [illegible]hort History of Electronic Examinations (E-exams)

According to Russell, Goldberg and C[illegible]nic exams began to be used in the late 1960s by the US Department of Defen[illegible]nce on clinical psychologists, who are considered the pioneers of computer[illegible]ns. During that period, a number of researchers conducted studies compari[illegible]per exams, with the aim of determining the efficiency, validity, and reliability of electronic exams on measures of personality and

Literature Review is very tall, need to summarize, then to add topic about Education challenges and exams during the Corona period

intelligence (Morris, Brandsford & Franks, 1977). Furthermore, Ćwil (2019) pointed out that the emergence of the idea of electronic exams began with the advent of the first computers in the 1970s, whenever the ability of this new technology to create entirely new environments for the design and management of the exams was acknowledged. Moreover, the idea and feasibility of electronic exams was developed significantly in the 1990s with the widespread diffusion of the Internet, which facilitated communication processes and helped to establish electronic exams as a way of assessing and evaluating students electronically. Russell et al. (2003) pointed out that electronic exams were not used in the field of education before the 1980s, but from this time, studies comparing electronic and paper exams, and identifying factors that affect users' performance of electronic exams, began to emerge. Akdemir and Oguz (2008) indicate that the use of computers and the internet for evaluation purposes in higher education institutions around the world increased widely and rapidly from the beginning of the twenty-first century. Some national governments have shown a trend of adopting computerized exams in various stages of public education and higher education. For example, the UK government began to adopt electronic exams in public education from 2008 (Nugent, 2003). Moreover, Hillier (2014) indicates that electronic exams are currently being used in a wider range of areas, such as Medical Licencing Examination (USMLE MCQ), Microsoft Certified Systems Engineer (MCSE), Cisco Certifications and The International English Language Testing System (IELTS). Nowadays, during the spread of COVID-19, most educational institutions are switching to electronic exams conducted online. This is due to the fact that electronic exams are safer for students and teachers during the COVID-19 pandemic, they will save time for lecturers, and they facilitate ease of planning and implementation, announcement, and analysis of results. Faculty members in universities and teachers in schools thus have the ability to prepare and implement electronic exams to measure the extent of their students' understanding of the course content and the extent to which learning objectives are achieved.

2.2 [illegible]finition of Electronic Exams (E-exams)

Electronic exams can be defined as all forms of assessment and evaluation that are carried out using digital technologies (Eltahir, Al-Qatawneh, Al-Ramahi, & Alsalhi, 2019; Nguyen, Rienties, Toetenel, Ferguson, & Whitelock, 2017). It is a process that includes the implementation of exams through the internet or intranet (Ayo, Akinyemi, Adebiyi, & Ekong, 2007). Zaitoun (2005) and

Farzin (2016) defined electronic exams as computer-based and internet-based, where questions are posed to students, corrected directly, with feedback provided on the student's responses and scores reported, and appropriate security measures taken to maintain confidentiality. Moreover, Sindre, and Chirumamilla (2015) defined e-exams as a timed, controlled, summative evaluation exam carried out using each candidate's own device working a unified operating system. According to Wibowo, Grandhi, Chugh, and Sawir (2016), e-exams have benefits compared to traditional paper exams, such as that they may include new multimedia and be interactive, and software test elements that have greater validity for it, in addition to ease of labeling, being time-saving, minimizing managerial expenses, and achieving reductions in the cost of raw materials.

2.3 Benefits of Electronic Exams (E-exams)

There is a group of studies that highlight a number of the main benefits of online electronic exams, in addition to some noticeable challenges from the point of view of both teachers and students, when compared to traditional printed paper exams (Alsalhi, Eltahir, & Al-Qatawneh, 2019; Cook & Jenkins, 2010; Dreher, Reiners, & Dreher, 2011; Hodgson & Pang, 2012; James, 2016). These can be summarized as seen in Figure 1.

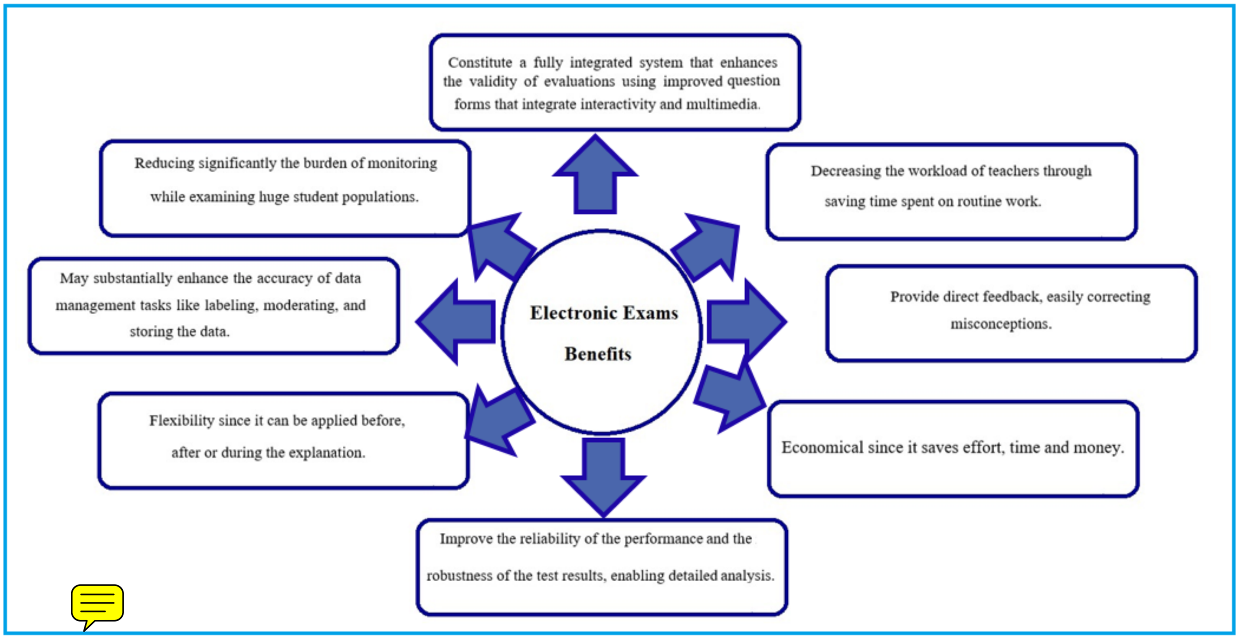

Figure 1. ***Benefits of electronic exams.***

Several studies pointed out that students prefer electronic exams over traditional printed exams because they can take the exam at any time and anywhere, they can obtain feedback and

marks more quickly, they have more control, are easy and quick to use, save time, and are environmentally friendly (Alzu'bi, 2015; Eljinini & Alsamarai, 2012; Marriott, 2009; Way, 2012; Williams & Wong, 2009).

In contrast, we find that traditional paper exams have numerous disadvantages, such as being time-consuming. Grading of paper-based exams needs to be done manually, which is difficult and laborious. The examination of many students requires more invigilators. Grades are not exact, as calculations are carried out manually. Examination results can be lost, and it takes a long time to check the results, as this is done manually.

2.4 Challenges Related to Electronic Exams During the COVID-19 Pandemic

After campus closures to curb the spread of COVID-19 pandemic, universities and schools have faced many challenges related to changing the system of learners' exams to electronic exams instead of traditional paper exams. According to the Organization for Economic Co-operation and Development (OECD, 2020), Alruwais, Wills, and Wald (2018), and Ryall, Judd, and Gordon (2016), there are many challenges to such implementation. These are summarized in Figure 2.

Possible student academic dishonesty

- In reality, this means and translates to cheating and plagiarism, which is by far the most widely debated problem in educational institutions today with regard to the change to electronic exams.

Assessing and certifying practical knowledge and skills

- In certain respects, the evaluations of learners cannot be based on written exams to evaluate the learned knowledge and skills, because it requires a direct evaluation of the real ability of learners to perform a specific task.

Ensuring fairness

- All the learners meet in the same room during on-site exams to take an exam under similar conditions (timing, material available, etc.). In contrast, learners do not benefit from the same working conditions when taking off-site online tests.

Risk of technical failure

- This means that good and efficient LMS servers and systems are needed to ensure students and faculty members have access to the Internet from their homes.

Figure 2. ***Challenges related to electronic exams during COVID-19 pandemic.***

2.5 Electronic Exam Usability

In reviewing the linked literature and studies, the authors found that research about the application of electronic exams in universities and schools remains insufficient to justify their success and effectiveness in education and learning as a replacement for traditional printed exams. There have been a few studies on the application of electronic exams in educational institutions such as universities and schools, which have all centered on the features and benefits of electronic exams (Baleni, 2015; Ćwil, 2019; Hameed & Abdullatif, 2017; Hodgson & Pang, 2012; IsauAdewole et al., 2018; James, 2016; Lim, Ong, Wilder-Smith, & Seet, 2006; Marius et al., 2016; Spivey & McMillan, 2014). Results from these studies focused on the perceptions of students and faculty members concerning the relative benefits, features, and challenges of electronic exams and their efficiency compared to traditional paper exams. The results showed that students' showed their openness to and acceptance of the electronic exams. They also confirmed that they prefer electronic computerized tests of the type of multiple-choice questions; in addition to a preference for the feature in electronic exams that enables them to re-sit the exam several times in order to improve their scores. Additionally, in Spivey and McMillan (2014), the results indicated that the application of electronic exams does not have a negative impact on students' grades and academic achievement, and features of electronic exams were appropriate and accepted by students (Spivey & McMillan, 2014). Wang (2016) reported that the system of electronic examinations could reduce the burden on teachers and enhance instructional quality. Also, studies have confirmed that electronic exams offer direct feedback to students and help improve learning in comparison with traditional paper exams (Crews & Curtis, 2010; Eljinini & Alsamarai, 2012; Marriott, 2009; Way, 2012).

On the other hand, some other studies have shown that students were upset about the inability to explain their responses and answers because of strict computer technology settings, which raised their stress and confusion during the exam (Betlej, 2013). At the same time, some studies have shown that students' readiness for type of test the need to complete, together with the consistency of the exam, eventually affects their academic results (Gewertz, 2013). According to Kim (2020), there are reasons for removing the time-limits imposed on electronic exams during the COVID-19 pandemic: it causes unnecessary pressure on students; problems with bandwidth and network connectivity can cause delays; timed exams measure speed, which is only weakly connected with comprehension; is not secure from fraud; and it might put an extra burden on

learners who really need learning accommodations. Moreover, Da'asin (2016) pointed out that while electronic exams might be a reliable tool to measure what they aim to measure, they may also raise learners' anxiety and tension levels and might makes it easier to cheat.

On the other hand, Eshet-Alkalai and Geri (2007) pointed out that learners preferred print to digital displays during their studying and exams, as reading electronic online exams may lead to more cognitive stress load on the reader compared to reading from print exams. King et al. (2009) also revealed that learners thought it easier to cheat on electronic examinations conducted online.

2.6. Significance of Study

- Better understanding of students' perspectives on e-exams will assist in identifying the major challenges in achieving undergraduate students' acceptance of their application.
- The findings of the current study might encourage and facilitate the sustained switchover to E-learning and electronic exams as an assessment process in the education system during and after COVID-19.
- Findings of the current study might benefit higher education institutions and other educational sectors outside of the United Arab Emirates.

3. Method

3.1 Approach of the Study

The current analysis was carried utilizing a descriptive method approach, which is a type of research that describes an under-examined population, condition, or phenomena by gathering quantifiable data that can be used for statistical analysis (Shields, Patricia, & Rangarajan, 2013). According to Nassaji (2015), the prime purpose of descriptive research is to examine phenomena and their specific features. Moreover, it deals with what really occurred, instead of why or how (Gall, Gall, & Borg, 2007). Thus, a questionnaire instrument was utilized to gather data from a sample of the population.

3.2 Population of Study

The research population comprised male and female students of all Ajman University colleges registered in the second semester in the academic year 2019/2020. The total number was 6620 undergraduate students as shown in Table 1 and Figure 3.

Table 1

Study Population

	College	# of students	(%)
**1**	College of Dentistry	944	14.3%
**2**	College of Pharmacy & Health Sciences	397	6.0%
**3**	College of Engineering and Information Technology	1209	18.3%
**4**	College of Architecture, Art and Design	531	8.0%
**5**	College of Business Administration	979	14.8%
**6**	College of Law	545	8.2%
**7**	College of Mass Communication	589	8.9%
**8**	Colleg[illegible] Humanities and Sciences	1304	19.7%
**9**	College of Medicine	122	1.8%
	**Total**	**6620**	**100.0%**

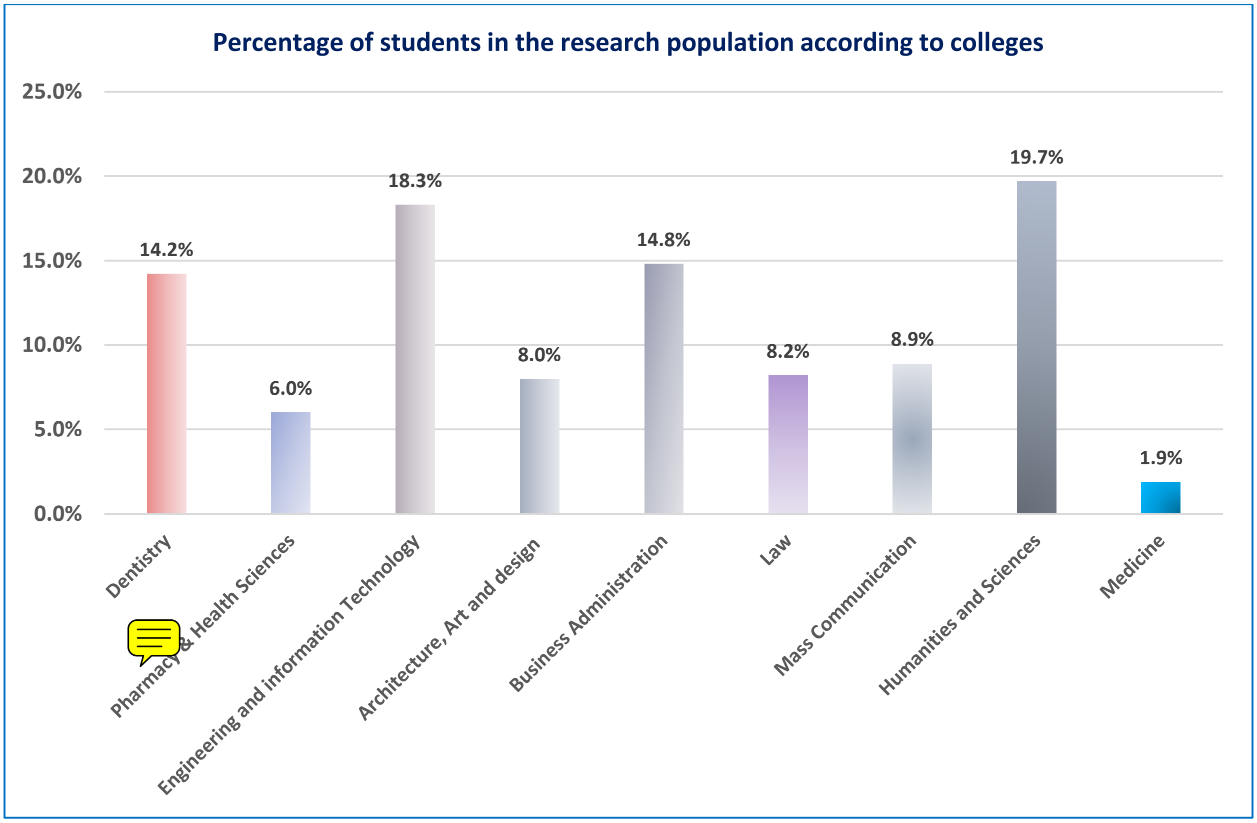

Figure 3. ***Research population.***

3.3 Sample

A sample of 30% of the population of each college was taken by the investigators. A random sampling method, implemented through a stratified sample technique, was used to obtain the sample for this study, which totaled 1986 (6620 * 30/100 = 1986) students. For example, for the students of the College of Dentistry, 944 * 30/100 = 283.2, which indicated that a sample of 283 students was required from this college. As a percentage of the total sample, College of Dentistry students were 283 /1986* 100 = 14.2%. The same process was followed for the other colleges (see Table 2).

Table 2

Research sample

	College	# of students	Percentage (%)
**1**	Dentistry	283	14.2%
**2**	Pharmacy & Health Sciences	119	6.0%
**3**	Engineering and Information Technology	363	18.3%
**4**	Architecture, Art and Design	159	8.0%
**5**	Business Administration	294	14.8%
**6**	Law	163	8.2%
**7**	Mass Communication	177	8.9%
**8**	Humanities and Sciences	391	19.7%
**9**	Medicine	37	1.9%
**Total**		**1986**	**100.0%**

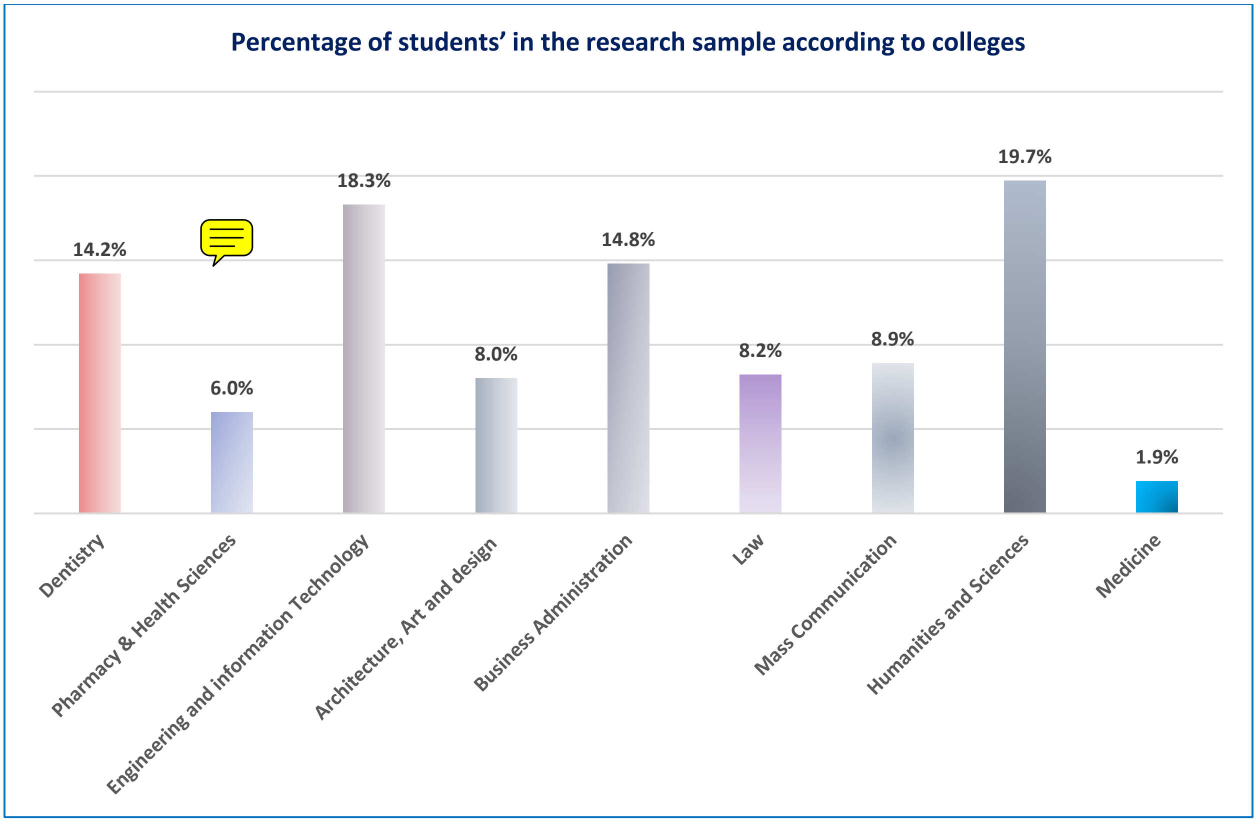

Figure 4. ***Research sample.***

A total of 1986 questionnaires were distributed to students in order to collect the data needed to achieve the study objectives. Of these, 1742 were returned completed correctly and in full. A number of learners (n=244) across all selected colleges did not responding correctly to the questionnaire. Consequently, the sample became 1742 students. Table 3 shows the demographic data for the selected sample of students who answered the questionnaire correctly.

Table 3

Demographic Information of Students

Study variables	Variables levels	Frequency (f)	Percentage (%)
**Gender**	Female	964	55.3%
	Male	778	44.7%
	**Total**	**1742**	**100.0%**
**College**	Dentistry	253	14.5%
	Pharmacy & Health Sciences	112	6.4%
	Engineering and Information Technology	295	16.9%

	Architecture, Art and Design	146	8.4%
	Business Administration	264	15.2%
	Law	161	9.2%
	Mass Communication	144	8.3%
	Humanities and Sciences	334	19.2%
	Medicine	33	1.9%
	**Total**	**1742**	**100.0%**
**Academic year**	First	542	31.1%
	Second	447	25.7%
	Third	285	16.4%
	Fourth	234	13.4%
	Fifth	234	13.4%
	**Total**	**1742**	**100.0%**

3.3 Study Instrument

The questionnaire was used to gather data from the sample learners. It was sent to them during the second semester of the academic year 2019/2020, during the occurence of the COVID-19 pandemic. During the design of the questionnaire, similar research in this area was reviewed, such as studies conducted by Hassan and Al Mari (2016) and Shraim (2019).

The questionnaire comprised of two sections. The first section concerned students' general information, and the second part represented the questionnaire elements (n=27) based on the study's objectives.

- **The validity of the instrument**

A group of arbitrators (10 faculty members of UAE universities) with extensive experience in the field of education were asked to express their views on the items of the questionnaire, in terms of the relevance of items for achieving the research aims and the number and comprehensiveness of the questionnaire items. The educational specialists' comments and suggested modifications were taken into account, and relevant deletions, amendments, and additions were made. As a result, the questionnaire after modification consisted of 27 elements, to achieve the objective of the research.

- **Reliability of the instrument**

To verify the internal consistency of the study tool, Cronbach's α was used. It was applied to a pilot study involving 50 students from outside the study sample, for which the calculated Cronbach alpha coefficient was 0.874.

3.4 Data Analysis Measures

In this analysis, a five-dimensional Likert scale is implemented, as shown in Table 4 below.

Table 4

Evaluation of Scale Data Based on the Options of Scale and Score Intervals

Description	Scores	Intervals
Very high	5	4.21–5.00
High	4	3.41–4.20
Moderate	3	2.61–3.40
Low	2	1.81–2.60
Very low	1	1.00–1.80

3.5 Statistical Analysis of the Data

For data analysis, the researchers utilized the Statistical Package for the Social Sciences (SPSS) to compute the percentage, mean, standard deviation SD, independent t-test tests, one-way ANOVA, and the Scheffe test.

4. Results

4.1 Findings of the study attributed to Question 1: *To what extent do Ajman University undergraduate students accept electronic exams during the spread of the COVID-19 pandemic?*

To address the first research question, we computed average scores and standard deviations of participants' responses to every one of the questions 1−27, which were relevant to the students' acceptance of electronic exams during the spread COVID-19 pandemic, as seen in Table 5.

Table 5

Descriptive Statistics for the Students' Responses to the Items about the Degree of Acceptance of Electronic Exams

No.	Paragraphs	Mean	SD	Description
Q1	[illegible]lectronic exams provide a more engaging experience than using paper	3.23	1.34	Moderate
Q2	Electronic exams are more environmentally friendly than paper exams	3.68	1.25	High
Q3	Electronic exams provide the ability to easily identify and access unanswered questions	3.62	1.09	High
Q4	I think that electronic exams are more familiar for me than printed paper exams	3.45	1.11	High
Q5	The number of electronic exam questions is sufficient	3.56	1.11	High
Q6	The electronic exam system is clear and specific	3.59	1.11	High
Q7	Electronic exams help extract results quickly, meaning feedback and marks are provided more quickly	4.13	1.00	High
Q8	Electronic exam regulations are clear and easy to understand	3.64	1.09	High
Q9	Electronic exam times are appropriate for students	2.68	1.17	Moderate
Q10	I think the electronic exam system was successful in protecting against technical problems	2.54	1.16	Moderate
Q11	Students do not need external help when using the computer [for electronic exams]	2.57	1.32	Moderate
Q12	The exam time is not enough to answer all questions	3.21	1.33	Moderate
Q13	Electronic exams help raise the efficiency of student achievement	3.01	1.33	Moderate
Q14	Electronic exams serve as an accurate and reliable assessment method	2.67	1.29	Moderate
Q15	I would recommend the electronic exams system to others	2.91	1.34	Moderate
Q16	Electronic exams are suitable for assessing students on any course	2.90	1.31	Moderate
Q17	Taking the electronic exam requires less time than taking the paper-based exam	2.87	1.25	Moderate
Q18	I prefer taking paper-based exams for assessing my knowledge	2.96	1.23	Moderate
Q19	Electronic exams enable me to show a better academic achievement	3.04	1.36	Moderate
Q20	Electronic exams serve as a flexible assessment method	3.00	1.33	Moderate
Q21	Electronic exams make me feel less stressed than paper-based exams	3.29	1.27	Moderate
Q22	I feel that the program (software) in the electronic exams system is easy to use and deal with	3.27	1.21	Moderate
Q23	In general, I prefer taking electronic exams to taking paper-based exams	2.94	1.22	Moderate
Q24	Internet interruption while I am doing electronic exams causes me great anxiety	3.72	0.99	High
Q25	Electronic online exams make me feel more stress, pressure, and anxiety compared to printed paper-based exams	3.15	1.30	Moderate
Q26	I feel that it is easy to cheat while performing electronic exams	3.70	1.03	High

Q27	I think that electronic exams are more difficult than traditional exams	2.56	1.32	Moderate
**Total**		**3.18**	**1.22**	Moderate

The findings shown in Table 5 show that the mean for responses for all items (1–27) was 3.18 (SD 1.22), indicating that the students showed a moderate acceptance of electronic exams during the COVID-19 pandemic. This finding might indicate that some students still prefer traditional paper exams, even though there is a COVID-19 pandemic.

It is also evident from Table 5 that the students' answers to Q-7 ('Electronic exams help extract results quickly, meaning feedback and marks are provided more quickly') was given the highest mean value (4.13) at a high degree, and Q-24 ('Internet interruption while I am doing electronic exams causes me great anxiety') came in second, also at a high level with a mean value of 3.72. Q-26 ('I feel that it is easy to cheat while performing electronic exams') came in third, at a high level with a mean value of 3.70. Moreover, Q-2 ('Electronic exams are more environmentally friendly than paper exams') came in fourth, also at a high degree of acceptance of electronic exams with a mean value of 3.68. Furthermore, it is also evident from the students' responses to Q-8 ('Electronic exam regulations are clear and easy to understand') that this question was rated as having the fifth highest degree of acceptance of using electronic exams, with a mean of 3.64, and at a high degree. Similarly, a high degree was also found for Qs 3, 4, 5, and 6 with the respective mean values of 3.62, 3.45, 3.56, and, 3.59.

The lowest mean (2.54) was acquired for Q-10 ('I think the electronic exam system was successful in protecting against technical problems'), suggesting a moderate degree. In the same way, a moderate degree also obtained for Qs 1, 9, 11, 12, 13, 14, 15, 16, 17, 18, 19, 20, 21, 22, 23, 25, and 27, with the respective mean values of 3.23, 2.68, 2.57, 3.21, 3.01, 2.67, 2.91, 2.90, 2.87, 2.96, 3.04, 3.00, 3.29, 3.27, 2.94, 3.15 and 2.56.

4.2 **Findings of the study attributed to Question 1:** *Does the degree Ajman University undergraduate students' acceptance of electronic exams during the spread of COVID-19 vary according to gender, college, and academic year?*

Mean scores and SD were calculated for questions, and t-test, one-way ANOVA test, and Scheffe's post-hoc comparison test were also conducted to determine the significance of the variations between averages. The findings of the answers to the study subjects are listed below according to the study variables.

First: Gender variations among students

A t-test was utilized to assess the significance of the differences between the averages of the acceptance of electronic exams by undergraduate students at Ajman University during the spread COVID-19, from the perspective of students, according to gender, as shown in Table 6.

Table 6

Means and SD of the Students' Answers Based on Gender

Gender	N	Mean	SD	Mean Difference	T. Value	df	Sig.
**Female**	42	3.06	.503	0.05814	2.037	1740	0.042*
**Male**	45	3.20	.723				
*** Statistically significant at (p<0.05)**							

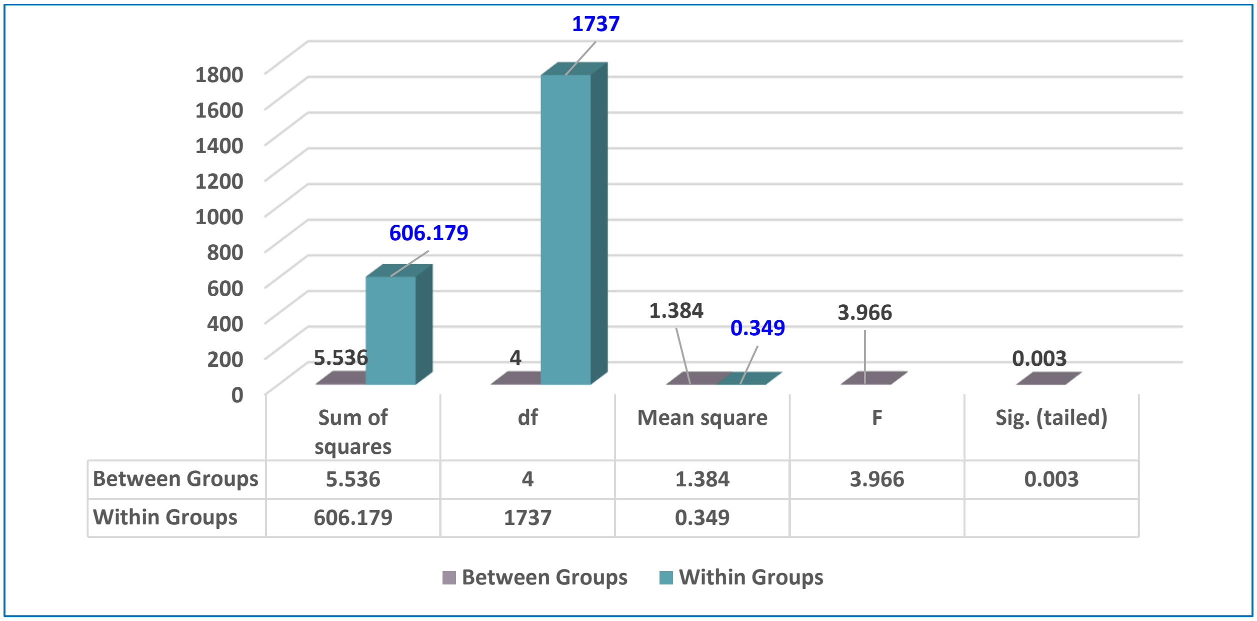

Figure 5. ***Means and standard deviations of the students' answers based on gender.***

As presented in Table 6 and Figure 5, the findings clearly illustrated that the computed t value was 2.037, which is greater than the (t) table, indicating the presence of significant differences between the mean values for males and females (in favor of females), at the significance level of 0.042, which is less than the required statistical significance level (0.05). The

finding means that female Ajman University undergraduate students are more accepting than their male counterparts of electronic exams during the spread of COVID-19.

Second: College variable among students

A one-way ANOVA test was utilized to assess the significance of the differences between averages of Ajman University undergraduate students' acceptance of electronic exams during the spread COVID-19, according to college variable among students. The findings of the one-way ANOVA test of this variable are shown in Table 7 and Figure 7. As displayed in Table 7 and Figure 6, the results clearly illustrated that there are statistically significant differences in students' perspectives according to the variable of college, as the p-value is 0.003, which is less than the required statistical significance level (0.05).

Table 7

One-way ANOVA test for College Variable Among Students

		Sum of squares	df	Mean square	F	Sig. (tailed)	Sig. level
College variable	**Between Groups**	16.394	8	2.049	5.966	0.001	Significant
	**Within Groups**	595.321	1733	0.344			
	**Total**	611.715	1741				

* Statistically significant at ($p<0.05$)

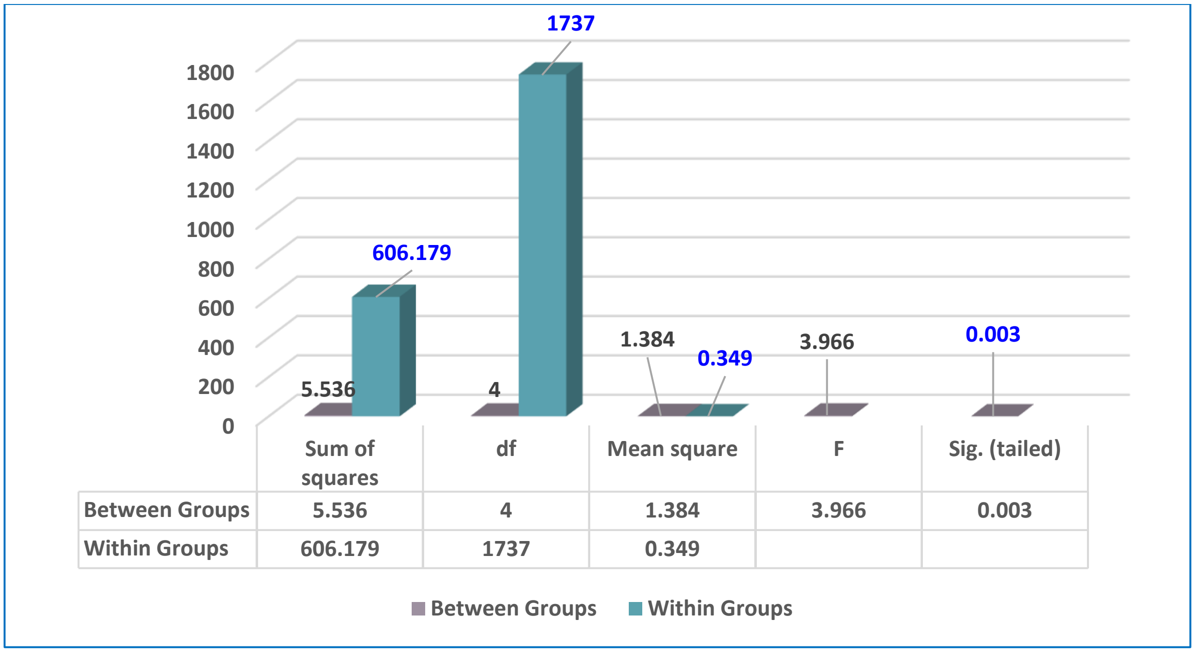

Figure 6. ***One-way ANOVA test for college variable among students.***

Therefore, in order to identify the source of the differences, the Scheffe test was used for the following comparisons, and the findings are shown in Table 8 below. The results shown in Table 8 emphasize that the source of the differences in the students' acceptance of electronic exams according to the variable of college was in favor of students of the Pharmacy & Health Science College.

Table 8

The Scheffe Test Results According to the College Variable

(I) The college	(J) The college	Mean difference (I-J)	Sig.
Dentistry	Pharmacy & Health Science	.15351	.722
	Business Administration	-.01737	1.000
	Engineering and Information Technology	-.15540	.297
	Architecture, Art and Design	-.05822	.999
	Law	-.23680*	.042
	Mass and Communication	.03189	1.000
	Humanities and Sciences	.00623	1.000
	Medicine	-.03308	1.000
Pharmacy &Health Science	Dentistry	-.15351	.722
	Business Administration	-.17089	.571
	Engineering and Information Technology	-.30892*	.004

	Architecture, Art and Design	-.21174	.408
	Law	-.39031*	.000
	Mass Communication	-.12162	.951
	Humanities and Sciences	-.14728	.725
	Medicine	-.18660	.958
Business Administration	Dentistry	.01737	1.000
	Pharmacy & Health Science	.17089	.571
	Engineering and Information Technology	-.13803	.461
	Architecture, Art and Design	-.04085	1.000
	Law	-.21943	.082
	Mass Communication	.04927	1.000
	Humanities and Sciences	.02360	1.000
	Medicine	-.01571	1.000
Engineering and Information Technology	Dentistry	.15540	.297
	Pharmacy & Health Science	.30892*	.004
	Business Administration	.13803	.461
	Architecture, Art and Design	.09718	.952
	Law	-.08139	.981
	Mass Communication	.18730	.274
	Humanities and Sciences	.16163	.156
	Medicine	.12232	.996
Architecture, Art and Design	Dentistry	.05822	.999
	Pharmacy & Health Science	.21174	.408
	Business Administration	.04085	1.000
	Engineering and Information Technology	-.09718	.952
	Law	-.17858	.525
	Mass Communication	.09012	.989
	Humanities and Sciences	.06445	.996
	Medicine	.02514	1.000
Law	Dentistry	.23680*	.042
	Pharmacy & Health Science	.39031*	.000
	Business Administration	.21943	.082
	Engineering and Information Technology	.08139	.981
	Architecture, Art and Design	.17858	.525
	Mass Communication	.26869*	.043
	Humanities and Sciences	.24303*	.017
	Medicine	.20371	.913
Mass Communication	Dentistry	-.03189	1.000
	Pharmacy & Health Science	.12162	.951
	Business Administration	-.04927	1.000
	Engineering and Information Technology	-.18730	.274
	Architecture, Art and Design	-.09012	.989
	Law	-.26869*	.043
	Humanities and Sciences	-.02566	1.000
	Medicine	-.06498	1.000
Humanities and Sciences	Dentistry	-.00623	1.000
	Pharmacy & Health Science	.14728	.725
	Business Administration	-.02360	1.000

	Engineering and Information Technology	-.16163	.156
	Architecture, Art and Design	-.06445	.996
	Law	-.24303*	.017
	Mass Communication	.02566	1.000
	Medicine	-.03932	1.000
Medicine	Dentistry	.03308	1.000
	Pharmacy & Health Science	.18660	.958
	Business Administration	.01571	1.000
	Engineering and Information Technology	-.12232	.996
	Architecture, Art and Design	-.02514	1.000
	Law	-.20371	.913
	Mass Communication	.06498	1.000
	Humanities and Sciences	.03932	1.000
* Statistically significant at (p<0.05)			

Third: Academic year variable

A one-way ANOVA test was utilized to assess the significance of the differences between averages of the acceptance of electronic exams by undergraduate students at Ajman University during the spread COVID-19, according to academic year variable. The findings of the one-way ANOVA test of this variable are shown in Table 9 and Figure 8. As displayed in Table 9 and Figure 7, the results clearly illustrate that there are statistically significant differences in students' perspectives according to the variable of academic year, as the p-value is 0.003, which is less than the required statistical significance level (0.05).

Table 9

One-way ANOVA Test for Academic Year Variable Among Students

		Sum of squares	df	Mean square	F	Sig. (tailed)	Sig. level
Academic year	**Between Groups**	5.536	4	1.384	3.966	0.003*	Significant
	**Within Groups**	606.179	1737	.349			
	**Total**	611.715	1741				
* Statistically significant at (p<0.05)							

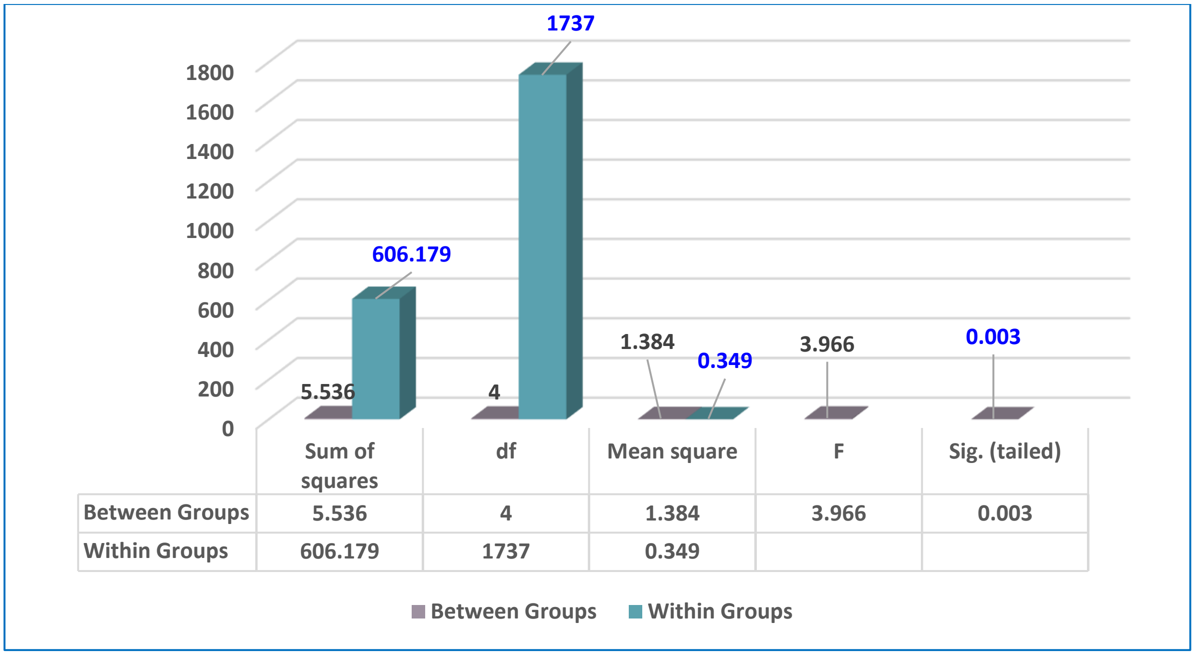

Figure 7. ***One-way ANOVA test for academic year variable among students.***

Therefore, in order to identify the source of the differences, the Scheffe test was used for the following comparisons, and the findings are shown in Table 10 below. The results shown in Table 10 indicate that the source of the differences in the students' acceptance of electronic exams according to the variable of academic year was in favor of students in their third academic year.

Table 10

The Results of the Scheffe Test According to the Academic Year Variable

(I) Academic Year	Mean Difference (I-J)	Sig.	(I) Academic Year
First	Second	.03336	.922
	Third	-.08773	.253
	Fourth	.11649	.289
	Fifth	.31250	.968
Second	First	-.03336	.922
	Third	-.12109	.077
	Fourth	.08313	.683
	Fifth	.27914	.979
Third	First	.08773	.253
	Second	.12109	.077
	Fourth	.20422*	.012

	Fifth	.40023	.923
Fourth	First	-.11649	.289
	Second	-.08313	.683
	Third	-.20422*	.012
	Fifth	.19601	.995
Fifth	First	-.31250	.968
	Second	-.27914	.979
	Third	-.40023	.923
	Fourth	-.19601	.995
* Statistically significant at (p<0.05)			

5. Discussion

Results of the first research question on the degree of acceptance of electronic exams during the spread of the COVID-19 pandemic at Ajman University indicate that, from the students' own perspective, the degree of acceptance of electronic exams was at a moderate level, with a general arithmetic mean of 3.18 and standard deviation of 1.22. The moderate result might mean that some of the undergraduate students at Ajman University accepted the implementation of electronic exams during the COVID-19 pandemic, while others did not, preferring the traditional paper-based exams.

Based on the results in Table 5, related to the students' responses to the questionnaire items, it was noted that some of their responses indicated positive attitudes towards the implementation of electronic exams at Ajman University during the Covid-19 pandemic. Items Q-2, Q-3, Q-4, Q-5, Q-6, Q-7 and Q-8 all indicated high degrees of acceptance. This means that undergraduate students might be satisfied to accept the implementation of electronic exams in their university during the Covid-19 pandemic spread, which may be due to reasons related to the features of electronic exams such as quicker feedback and marks, saving time, environmentally friendly, easy to identify and access unanswered questions, the system of electronic exams being clear and easy, and the ability of learners to take the exam anywhere and at any time. These results are consistent with those of previous studies (Baleni, 2015; Ćwil, 2019; Hameed & Abdullatif, 2017; Hodgson & Pang, 2012; IsauAdewole et al. 2018; James, 2016; Lim, Ong, Wilder-Smith, & Seet, 2006; Marius et al., 2016; Spivey & McMillan, 2014). The results of these studies indicated that students showed their openness to and acceptance of electronic exams, and they also confirmed that they prefer electronic exams, especially when the type of questions are multiple-

choice or true/false. Moreover, the results agree with studies that confirmed that students prefer electronic exams because they provide marks and feedback more quickly, and help them to improve their learning and understanding of the content of the curriculum compared to traditional paper exams (Crews & Curtis, 2010; Eljinini & Alsamarai, 2012; Marriott, 2009; Way, 2012).

In contrast, however, some of the students' responses to the questionnaire items showed negative attitudes towards the implementation and application of electronic exams at their university during the Covid-19 pandemic spread. For example, they responded to Q-24 ('Internet interruption while I am doing electronic exams causes me great anxiety') with a high level, with a mean value of 3.72. This means that undergraduate students at Ajman University might be feeling anxiety and stress as a result of carrying out electronic exams rather than traditionally printed examination papers. This result is consistent with previous studies that found that students were upset, confused, and nervous about their inability to explain their responses and answers during electronic exams, due to strict computer technology settings (Betlej, 2013; Gewertz, 2013; Kim, 2020).

Furthermore, Ajman University undergraduate students' responses to Q-26 ('I feel that it is easy to cheat while performing electronic exams') also came with a high level, with a mean value of 3.70. This might mean that undergraduate students at Ajman University might feel that there may be opportunities for some cases to cheat while students perform electronic exams. This result may be consistent with the study conducted by King et al. (2009), who pointed out that learners thought it easier to cheat when doing electronic examinations online. Also, it is consistent with the results obtained by Da'asin (2016), who pointed out that electronic exams might be a reliable and competent tool to measure what they aim to measure, but they may raise learners' anxiety and tension levels and might make cheating easier.

Moreover, Ajman University undergraduate students' responses to Q-10 ('I think the electronic exam system was successful in protecting against technical problems') came with the lowest mean value of 2.54. This might mean that undergraduate students at Ajman University might have faced some technical problems while taking their electronic exams during the spread COVID-19 pandemic through the second semester of the academic year 2019/2020. This result might be consistent with the studies conducted by Betlej (2013) and Kim (2020), who pointed out

that learners were upset and confused about not being able to answer electronic exams questions due to strict computer technology settings.

The second research question focused on determining whether the degree of acceptance of electronic exams by undergraduate students at Ajman University during the spread COVID-19 varied, from the students' perspectives, according to gender, college, and academic year. Our findings (as seen in Tables 6, 7, 8, 9, and 10, and Figures 5, 6, and 7) showed that the degree of acceptance of electronic exams by undergraduate students varies according to gender in favor of females. This result means that female Ajman University undergraduate students' acceptance of electronic exams during the spread COVID-19 is greater than the acceptance of their male counterparts. Also, the results indicate that acceptance also varies according to college type (in favor of the Pharmacy & Health Science College), and according to academic year (in favor of the third academic year).

6. Conclusion

Due to the spread of the COVID-19 pandemic, most educational institutions, such as universities and schools, have moved towards using technology in the process of assessing students through the implementation of electronic exams during the learning and teaching process. It might that a large-scale shift towards electronic exams can be expected during the next few years if the COVID-19 pandemic is not completely controlled in the world.

The current study aimed to explore the acceptance of electronic exams on the part of Ajman University undergraduate students during the spread of the COVID-19 pandemic. Findings showed that Ajman University undergraduate students demonstrated a moderate acceptance of electronic exams during the spread COVID-19 pandemic, with a total average and SD respectively of 3.18 and 1.22. This may indicate that some students accepted electronic exams and they gave a positive impression about it in their responses to the items of the questionnaire. This impression might due to the positive advantages that are distinguished by them, such as quicker feedback and marks, time savings, environmentally friendly, easy to identify and access unanswered questions, the system of electronic exams being clear and easy, and the ability of learners to take the exam anywhere and at any time. At the same time, responses of the undergraduate students to some other questionnaire items showed negative attitudes towards the implementation and application of electronic exams at their university during the Covid-19 pandemic. This impression might due to

their feelings of anxiety and stress as a result of completing electronic exams rather than traditionally printed examination papers. Also, it may be due to the fact that they were confused and nervous about their inability to explain their responses and answers during the electronic exam, due to strict computer technology settings.

Furthermore, the findings indicated that female Ajman University undergraduate students' acceptance of electronic exams during the spread COVID-19 is greater than the acceptance of their male counterparts. Also, the results indicate that acceptance also varies according to college type (in favor of the Pharmacy & Health Science College), and according to academic year (in favor of the third academic year).

7. Limitations of the Study

Like any other analysis, this study has some limitations that should be acknowledged.

First, this study was limited to students' responses, and the responses of faculty members were not taken. Second, the study was limited to a sample size of 1986 students, representing 30% of the study population.

8. Implications and Recommendations

Notwithstanding the aforementioned limitations, the following suggested educational implications and recommendations are provided for future research on the implementation of electronic exams during the spread of the COVID-19 pandemic.

- Most higher education institutions adopted the decision to temporarily avoid all in-person contact and close their campuses completely during the COVID-19 pandemic, which led these institutions to apply online electronic exams instead of traditional paper exams. Thus, it necessary to provide students with accurate and fair grades. This requires universities to provide a protection systes for these electronic exams on an ongoing basis.
- Appropriate solutions need to be found to technical problems and the disruption of the internet during the implementation of electronic exams.
- It is necessary to provide technical support on an ongoing basis while conducting electronic tests.
- It is necessary that processes be established to ensure that there are no cases of cheating during electronic examinations.

- Similar research should be performed on the implementation of electronic exams in higher education institutions.

9. Delimitations of the study

- *Location Limit*: Ajman University, Main campus, Ajman, UAE.
- *Time Limit*: Second semester of the academic year 2019/2020.
- *Human Limit*: The students of Ajman University in all colleges.

Acknowledgements

The authors' would like to thank Ajman University for its cooperation, and the Dean of Scientific Research, for his guidance and mentorship.

Availability of data and materials

While we would have liked to share the data, it is primary, and sharing it would therefore pose a risk of breaches of participant confidentiality.

Funding

This research received no specific grant from any funding agency in the public, commercial, or not-for-profit sectors. (There was no funding for this paper.)

10. References

Abass, Olalere A., Olajide, Samuel A., & Samuel, Babafemi O. (2017). Development of web-based examination system using open source programming model. *Turkish Online Journal of Distance Education-TOJDE,* 18(2), 30-42.

Akdemir, O., & Oguz, A. (2008). Computer-Based Testing: An Alternative for the Assessment of Turkish Undergraduate Students. *Computers & Education*, 51(3), 1198-1204.

Alruwais, N., G. Wills and M. Wald (2018), "Advantages and challenges of using e-assessment", *International Journal of Information and Education Technology*, Vol. 8/1, pp. 34-37.

Alsalhi, N., Eltahir, M., & Al-Qatawneh, S. (2019). The effect of blended learning on the achievement of ninth grade students in science and their attitudes towards its use. Heliyon, 5(9), e02424. https://doi.org/10.1016/j.heliyon.2019.e02424

Alzu'bi, M. (2015). The effect of using electronic exams on students' achievement and test takes motivation in an English 101 course. *Conference of the International Journal of Arts & Sciences*, 08(03):207–215

Ayo, C. K., Akinyemi, I. O.; Adebiyi, A. A. & Ekong, U. O. (2007). The prospects of e-examination implementation in Nigeria. *Turkish Online Journal of Distance Education*, 8(4), 125-134.

Betlej, P. (2013). E-examinations from student's perspective – The future of knowledge evaluation. *Studia Ekonomiczne*, 152, 9–22.

Baleni, Z. (2015). Online formative assessment in higher education: Its pros and cons. *The Electronic Journal of e-Learning*, 13(4), 228-236.

Cook, J. & Jenkins, V. (2010). Getting Started with e-Assessment. University of Bath. Accessed [illegible]ril 13, 2020 from https://www.researchgate.net/publication/336149217_Online_Examination_Practices_in_Higher_Education_Institutions_Learners'_Perspectives

Crews, T and Curtis, D. (2010). Online course evaluations: Faculty perspective and strategies for improved response rates. *Assessment & Evaluation in Higher Education*, 36 (7), 965–878.

Ćwil, M. (2019). Teacher's Attitudes towards Electronic Examination - a Qualitative Perspective. *International Journal of Learning and Teaching*, 5 (1), 77- 82.

Dreher, C., Reiners, T., & Dreher, H. (2011). Investigating Factors Affecting the Uptake of Automated Assessment Technology. *Journal of Information Technology Education*, 10, 161-181.

Da'asin, K. (2016). Attitude of Ash-Shobak University College Students to E-Exam for Intermediate University Degree in Jordan. *Journal of Education and Practice*. 7(9).10-17.

Eljinini, M and Alsamarai, S. (2012). The impact of e-assessments system on the success of the implementation process. *Modern Education and Computer Science*, 4(11), 76–84.

Eltahir, M., Al-Qatawneh, S., Al-Ramahi, N., Alsalhi, N. (2019). The perspective of students and faculty members on the efficiency and usability of E-learning courses at Ajman university: A case study. Journal of Technology and Science Education. 9(3): 388-403.

Eshet-Alkalai, Y., Geri, N. (2007). Does the Medium Affect the Message? The Influence of Text Representation Format on Critical Thinking. *Human Systems Management*, 26(4), 269–279.

Farzin, S. (2016). Attitude of Students towards E-Examination System: an Application of E-Learning. *Management Science and Information Technology*, 1(2), 20-25.

Gall, M. D., Gall, J. P., & Borg, W. R. (2007). *Educational research*: An introduction (8th ed.). Boston: Pearson.

Gewertz, [illegible]2013). Transition to online testing sparks concerns. Accessed June 20, 2020. https://www.edweek.org/ew/articles/2013/10/30/10pencil_ep.h33.html.

Giannini[illegible] S. Jenkins and J. Saavedra. (2020). Reopening schools: When, where and how? UNESCO. Accessed June 16, 2020, from https://en.unesco.org/news/reopening-schools-when-where-and-how

Hameed, M,. Abdullatif, F. (2017). Online Examination System. International Advanced Research Journal in Science, *Engineering and Technology*, 4 (3), 106- 110.

Hassan, M, and Al Mari, M. (2016). Attitudes of faculty members and students of the College of Education at Najran University towards electronic testing. *Journal of Gulf and Arabian Peninsula Studies*, 163(42), 17-51.

Hillier, M[illegible]014). *E-Exams with student owned devices: Student voices*. Proceedings of the International Mobile Learning Festival, Hong Kong SAR China.

Hodgson, P., & Pang, M. Y. C. (2012). Effective formative e-assessment of student learning: a study on a statistics course. *Assessment & Evaluation in Higher Education*, 37(2), 215–225.

IsauAdewole, A., Olugbenga, A., Olusegun, A., and Susan, K. (2018). Students' Perception of Computer-Based Examinations: A Case Study of Ladoke Akintola University of Technology, Ogbomoso Oyo State, Nigeria. *Journal of Humanities and Social Science*. 23(5), 1-7.

James, R. (2016). Tertiary student attitudes to invigilated, online summative examinations. *International Journal of Educational Technology in Higher Education*, 13(19), 1-13.

Kim, J. (2020). 5 Reasons to Stop Doing Timed Online Exams During COVID-19. Retrieved October 10, 2020. https://www.insidehighered.com/blogs/learning-innovation/5-reasons-stop-doing-timed-online-exams-during-covid-19

King, C.G., R.W. Guyette Jr, and C. (2009). Piotrowski, Online Exams and Cheating: An Empirical Analysis of Business Students' Views. *Journal of Educators Online*, 6(1), 1-11.

Lim, E., Ong, B., Wilder-Smith, E., & Seet, R. (2006).Computer-based versus pen-and-paper testing: students' perception. *Annals of the Academy of Medicine*. 35(9), 599-603.

Marriott, P. (2009). Students' evaluation of the use of online summative assessment on an undergraduate financial accounting module. *British Journal of Educational Technology*, 40 (2), 237–254.

Marius, P., Marius, M., Dan, S., Emilian, C., & Dana, G. (2016). Medical students' acceptance of online assessment systems. *Acta Medica Marisiensis*, 62(1), 30–32.

Nguyen, Q, Rienties, B,. Toetenel, L Ferguson, R and D. Whitelock, D. (2017). Examining the designs of computer-based assessment and its impact on student engagement, satisfaction, and pass rates. *Computers in Human Behavior*, vol. 76, 703-714.

Nugent, G. (2003) On-line multimedia assessment for K-4 students. Proceedings of the World *Conference on Educational Multimedia, Hypermedia and Telecommunications*, Hawaii, USA, 1051–1057.

Organisation for Economic Co-operation and Development OECD. (2020*). Remote online exams in higher education during the COVID-19 crisis*. OECD Education Policy Perspectives, No. 6, OECD Publishing, Paris.

Russell, M., Goldberg, A., & O'Connor, K. (2003). Computer-based testing and validity: A look back into the future. *Assessment in Education: Principles, Policy & Practice*, 10(3), 279-293.

Ridgway, [illegible]cCusker, S, and D. Pead. (2004). Literature Review of E-assessment,

Accessed September 5, 2020, from https://telearn.archives-ouvertes.fr/hal-00190440

Ryall, T., B. Judd and C. Gordon (2016), "Simulation-based assessments in health professional education: A systematic review", *Journal of Multidisciplinary Healthcare*, Vol. 9, 69-82.

Salem, Ahmed. (2004). *Education technology and E-learning, First edition*, Riyadh, Al-Rshed Library.

Shields, [illegible]icia, and Rangarajan, N. (2013). *A Playbook for Research Methods: Integrating Conceptual Frameworks and Project Management*. Stillwater, OK: New Forums Press.

Sindr[illegible]. and Chirumamilla, A. (2015). E-exams versus paper exams: A comparative analysis of cheating-related security threats and countermeasures. *Norwegian Information Security Conference (NISK)*. Retrieved August 19, 2020, from https://ojs.bibsys.no/index.php/NISK/article/view/298

Spivey, M. F., & McMillan, J. J. (2014). Classroom versus online assessment. *Journal of Education for Business*, 89, 450–456.

Sutton, R. E. (2004). Teaching under high-stakes testing: Dilemmas and decisions of a teacher educator. *Journal of Teacher Education*, 55(5), 463-475.

SWeaver, D., [illegible]al. (2005). Evaluation: WebCT and the student experience. *Evaluations and Assessment Conference.*

Wang, G. (2016). Design of a Student's Online Examination System Based on B/S Architecture. Advances in Social Science, *Education and Humanities Research (ASSEHR)*, volume 75, 181- 183.

Way, A. (2012). The use of e-assessments in the Nigerian higher education system. *Turkish Online Journal of Distance Education,* 13 (1), 140–152.

Williams, J and Wong, A. (2009). The efficacy of final examinations: A comparative study of closed-book, invigilated exams and open-book, open-web exams. *British Journal of Educational Technology*, 40 (2), 227–236.

Wibowo, S., Grandhi, S., Chugh, R. & Sawir, E. (2016). A Pilot Study of an Electronic Exam System at an Australian University. *Journal of Educational Technology Systems, 45*(1), 5-33.

Zaitoun, H. (2005). *New vision in e-learning - concept issues - implementation - evaluation*. Riyadh, the Solitary House of Education.
