# Supplementary material for: Implementation of E-exams during the COVID-19 pandemic: A quantitative study in higher education
Source: PLoS One. 2022 May 20;17(5):e0266940. doi: 10.1371/journal.pone.0266940 (PMC9122221; doi:10.1371/journal.pone.0266940)
Supplement: Attachment — Submitted filename: Modify some terms.docx [file pone.0266940.s003.docx]

1- Modify some terms:

| Electronic Exam | e-exam |
| --- | --- |
| during the spread of the COVID-19 pandemic | during the COVID-19 pandemic |
|  |  |

2- The faculty's attitudes toward e-exams were discussed in former research. The authors should explain why faculty's attitudes of using e-exams considered to be explored again during the COVID-19 pandemic.
3- The researchers did not take the influence of criteria for which the e-exam were designed, as well as the capabilities of the faculty members in designing the e-exam (especially since they are from specializations that do not have an electronic reference).
4- Research questions, that drive the paper, should be built in the introduction from an ongoing and pertinent bibliography (up to 2021). These should be of global interest and not focused to a particular local problem. Identifying a research gap is not enough; key is showing its significance to the field. It is better to get recent studies during the Corona period, because there are a large number of studies that have been done and dealt with axes close to the topic of research.
5- Answer your research question in the conclusions; what did we learn compared with current, significant research (up to 2021). The authors should make explicit suggestions about how their study affects the design or use of E-exam systems. Is there something new about a particular theory, or is there evidence of theory advancement?
6- How general are your results? These have to be of interest to the whole community. Relate these with your limitations. Please review the concept of research limitations such as history effects, sampling, etc.
7- Finally, what is the originality of this study? The authors should clarify the originality compared with other studies.
8- Explain how this paper differs from the related ones published in the technical literature.
9- - All references before 2010 are better to be replaced by modern references, especially in the discussion part of the search results.
10- Literature Review is very tall, need to summarize, then to add topic about Education challenges and exams during the Corona period.
11- It is preferable that the data be processed after dividing it based on the students’ specializations into three groups (humanitarian track – engineering track – health track) and each group is processed separately, because the specialization and related tasks can have a direct and significant impact.
12- The resolution for Figure 1. is very low.
13- There is no need for a diagram (figure 3 and 4) because it does not add anything new (just repeating) all the data are in the table (3 and 2).
14- The researchers did not clarify which program was used in designing the e-exam, and whether the same program was used, or whether each college used what it sees.
15- The researchers did not clarify whether the questionnaire was electronic or paper, and how it was distributed (whether by e-mail or otherwise).
