# Supplementary material for: Implementation of E-exams during the COVID-19 pandemic: A quantitative study in higher education
Source: PLoS One. 2022 May 20;17(5):e0266940. doi: 10.1371/journal.pone.0266940 (PMC9122221; doi:10.1371/journal.pone.0266940)
Supplement: Attachment — Submitted filename: Response to Reviewers..docx [file pone.0266940.s004.docx]

Dear Academic Editor, Christine E. King,

PLOS ONE Journal

Monday, January 31, 2022

PONE-D-21-20789

Subject: Revision and resubmission of manuscript (Manuscript Number: PONE-D-21-20789)

Manuscript title: *Implementation of E-exams During the COVID-19 Pandemic: A quantitative Study in Higher education*

***Dear Editor***

***First, I want to thank you and the reviewers for the extremely helpful comments provided for our paper. In the paper, I’ve addressed all comments – both specific and general –from the reviewers. We have implemented all the comments recommended.***

**Reviewer Comments, Authors Responses and Manuscript Changes**

**Reviewer #1:**

**Comment 1:**

Modify some terms:

| Electronic Exam | e-exam |
| --- | --- |
| during the spread of the COVID-19 pandemic | during the COVID-19 pandemic |
|  |  |

**Response:**

As the Reviewer suggested to us, we have modified the terms in the revised manuscript (*Kindly see the revised manuscript*).

**Comment 2**:

The faculty's attitudes toward e-exams were discussed in former research. The authors should explain why faculty's attitudes of using e-exams considered to be explored again during the COVID-19 pandemic.

**Response:**

The authors modified and explained this point in the revised manuscript as the reviewer suggested. (*Kindly see the revised manuscript*).

**After Modified**

**Comment 3**:

The researchers did not take the influence of criteria for which the e-exam were designed, as well as the capabilities of the faculty members in designing the e-exam (especially since they are from specializations that do not have an electronic reference).

**Response:**

The authors modified this point in the revised manuscript as the reviewer suggested. (*Kindly see the revised manuscript*)

**Comment 4**:

Research questions, that drive the paper, should be built in the introduction from an ongoing and pertinent bibliography (up to 2021). These should be of global interest and not focused to a particular local problem. Identifying a research gap is not enough; key is showing its significance to the field. It is better to get recent studies during the Corona period, because there are a large number of studies that have been done and dealt with axes close to the topic of research.

**Response:**

The authors obtained recent studies during the Corona period, and put them in the revised manuscript as the reviewer suggested to us (*Kindly see the revised manuscript*).

Examples of these recent studies carried out during the Corona period include (which we put them in the revised manuscript):

| **Recent studies conducted during the Corona period and which the authors added to the manuscript** | |
| --- | --- |
| 1 | Chirumamilla, A., & Sindre, G. (**2021**). E-exams in Norwegian higher education: Vendors and managers views on requirements in a digital ecosystem perspective. Computers & Education, 172 (1), 1-19. <https://doi.org/10.1016/j.compedu.2021.104263> |
| 2 | Khan, M.A., Vivek, V., Khojah, M., Nabi, M.K., Paul, M., &Minhaj, S.M. (**2021**). Learners’ Perspective towards E-Exams during COVID-19 Outbreak: Evidence from Higher Educational Institutions of India and Saudi Arabia. Int. International Journal of Environmental Research and Public Health. 18(12), 1-18. [https://doi.org/10.3390/ijerph18126534](%09https://doi.org/10.3390/ijerph18126534) |
| 3 | Liguori, E., & Winkler, C. (**2020**). From Offline to Online: Challenges and Opportunities for Entrepreneurship Education Following the COVID-19 Pandemic. Entrepreneurship Education and Pedagogy. 2020;3(4):346-351. doi:10.1177/2515127420916738 |
| 4 | [Ayoub/Al-Salim, M.I.](https://www.emerald.com/insight/search?q=Majda%20I.%20Ayoub/Al-Salim) & [Aladwan, K.](https://www.emerald.com/insight/search?q=Khaled%20Aladwan) **(2021**). The relationship between academic integrity of online university students and its effects on academic performance and learning quality. [*Journal of Ethics, Entrepreneurship and Technology*](https://www.emerald.com/insight/publication/issn/2633-7436), Vol. 1 No. 1, pp. 43-60. <https://doi.org/10.1108/JEET-02-2021-0009> |
| 5 | Raman, R., B, S., G, V., Vachharajani, H., & Nedungadi, P. (**2021**). Adoption of online proctored examinations by university students during COVID-19: Innovation diffusion study. *Education and information technologies*, 1–20. Advance online publication. https://doi.org/10.1007/s10639-021-10581-5 |
| 6 | Al-Darbashi, K. (**2021**). The Effectiveness of Using Online exams for Assessing Students In the human Sciences Faculties at the Emirati Private Universities during the COVID-19 crisis from their own perspective. *Review of International Geographical Education*, 11 (10), 1149-1160. |
| 7 | Gorgani, H. H., & Shabani, S. (**2021**). Online exams and the COVID-19 pandemic: a hybrid modified FMEA, QFD, and k-means approach to enhance fairness. SN applied sciences, 3(10), 818. <https://doi.org/10.1007/s42452-021-04805-z> |
| 8 | Kim, J. (**2020**). 5 Reasons to Stop Doing Timed Online Exams During COVID-19. Retrieved October 10, 2020. <https://www.insidehighered.com/blogs/learning-innovation/5-reasons-stop-doing-timed-online-exams-during-covid-19> |
| 9 | Holden, OL., Norris, ME., & Kuhlmeier, VA. (**2021**). Academic Integrity in Online Assessment: A Research Review. Frontiers in Education. 6,639814, 1-14. DOI: 10.3389/feduc.2021.639814 |
| 10 | Giannini, S., Jenkins, S., & Saavedra, J. (**2020**). Reopening schools: When, where and how? UNESCO. Accessed June 16, 2020, from <https://en.unesco.org/news/reopening-schools-when-where-and-how> |

**Comment 5**:

How general are your results? These have to be of interest to the whole community. Relate these with your limitations. Please review the concept of research limitations such as history effects, sampling, etc.

**Response:**

Done. As the reviewer suggested. (*Kindly see the revised manuscript*).

**Comment 6**: Explain how this paper differs from the related ones published in the technical literature.

**Response:**

Done. As the reviewer suggested. (*Kindly see the revised manuscript*).

**Comment 7**: All references before 2010 are better to be replaced by modern references, especially in the discussion part of the search results.

**Response:**

All references were replaced by modern references in the discussion part of the manuscript as the reviewer was suggest to us. (*Kindly see the revised manuscript*).

**Comment 8**: Literature Review is very tall, need to summarize, then to add topic about Education challenges and exams during the Corona period.

**Response:**

Done. As the reviewer suggested, the authors deleted the part (2.1 A Short History of E- examinations (E-exams). Also, added some education challenges and exams during the Corona period with update references during corona period

(*Kindly see the revised manuscript*

**Comment 9**: The resolution for Figure 1. is very low.

**Response:**

Done. As the reviewer suggested. (*Kindly see the revised manuscript*).

**Comment 10**: There is no need for a diagram (figure 3 and 4) because it does not add anything new (just repeating) all the data are in the table (3 and 2).

**Response:**

Done, the diagram (figure 3 and 4) are deleted as the reviewer suggested. (*Kindly see the revised manuscript*).

**Comment 11**: The researchers did not clarify which program was used in designing the e-exam, and whether the same program was used, or whether each college used what it sees

**Response:**

**Comment 12**: The researchers did not clarify whether the questionnaire was electronic or paper, and how it was distributed (whether by e-mail or otherwise).

**Response:**

As the reviewer suggested, the authors clarify that the questionnaire was electronic, and it was distributed through e-mail, WhatsApp, and social media. (*Kindly see the revised manuscript*).

**Reviewer #2:**

**Response:**

- The authors obtained recent studies during the Corona period and put them in the discussion section and literature review revised manuscript as the reviewer suggested to us (Kindly see the revised manuscript).

- Also, the authors added new articles and recent studies in the revised manuscript (Kindly see the revised manuscript).

- The authors made language editing for the manuscript.

Examples of these recent studies carried out during the Corona period include (which we put them in the revised manuscript):

| **Recent studies conducted during the Corona period and which the authors added to the manuscript** | |
| --- | --- |
| 1 | Chirumamilla, A., & Sindre, G. (**2021**). E-exams in Norwegian higher education: Vendors and managers views on requirements in a digital ecosystem perspective. Computers & Education, 172 (1), 1-19. <https://doi.org/10.1016/j.compedu.2021.104263> |
| 2 | Khan, M.A., Vivek, V., Khojah, M., Nabi, M.K., Paul, M., &Minhaj, S.M. (**2021**). Learners’ Perspective towards E-Exams during COVID-19 Outbreak: Evidence from Higher Educational Institutions of India and Saudi Arabia. Int. International Journal of Environmental Research and Public Health. 18(12), 1-18. [https://doi.org/10.3390/ijerph18126534](%09https://doi.org/10.3390/ijerph18126534) |
| 3 | Liguori, E., & Winkler, C. (**2020**). From Offline to Online: Challenges and Opportunities for Entrepreneurship Education Following the COVID-19 Pandemic. Entrepreneurship Education and Pedagogy. 2020;3(4):346-351. doi:10.1177/2515127420916738 |
| 4 | [Ayoub/Al-Salim, M.I.](https://www.emerald.com/insight/search?q=Majda%20I.%20Ayoub/Al-Salim) & [Aladwan, K.](https://www.emerald.com/insight/search?q=Khaled%20Aladwan) **(2021**). The relationship between academic integrity of online university students and its effects on academic performance and learning quality. [*Journal of Ethics, Entrepreneurship and Technology*](https://www.emerald.com/insight/publication/issn/2633-7436), Vol. 1 No. 1, pp. 43-60. <https://doi.org/10.1108/JEET-02-2021-0009> |
| 5 | Raman, R., B, S., G, V., Vachharajani, H., & Nedungadi, P. (**2021**). Adoption of online proctored examinations by university students during COVID-19: Innovation diffusion study. *Education and information technologies*, 1–20. Advance online publication. https://doi.org/10.1007/s10639-021-10581-5 |
| 6 | Al-Darbashi, K. (**2021**). The Effectiveness of Using Online exams for Assessing Students In the human Sciences Faculties at the Emirati Private Universities during the COVID-19 crisis from their own perspective. *Review of International Geographical Education*, 11 (10), 1149-1160.  . |
| 7 | Gorgani, H. H., & Shabani, S. (**2021**). Online exams and the COVID-19 pandemic: a hybrid modified FMEA, QFD, and k-means approach to enhance fairness. SN applied sciences, 3(10), 818. <https://doi.org/10.1007/s42452-021-04805-z> |
| 8 | Kim, J. (**2020**). 5 Reasons to Stop Doing Timed Online Exams During COVID-19. Retrieved October 10, 2020. <https://www.insidehighered.com/blogs/learning-innovation/5-reasons-stop-doing-timed-online-exams-during-covid-19> |
| 9 | Holden, OL., Norris, ME., & Kuhlmeier, VA. (**2021**). Academic Integrity in Online Assessment: A Research Review. Frontiers in Education. 6,639814, 1-14. DOI: 10.3389/feduc.2021.639814 |
| 10 | Giannini, S., Jenkins, S., & Saavedra, J. (2020). Reopening schools: When, where and how? UNESCO. Accessed June 16, 2020, from <https://en.unesco.org/news/reopening-schools-when-where-and-how> |
